# Urban drinking water security in Gujarat

**DOI:** 10.1007/s40847-020-00122-0

**Published:** 2021-02-18

**Authors:** Meera Mehta, Dinesh Mehta, Jaladhi Vavaliya

**Affiliations:** Centre for Water and Sanitation, CRDF, CEPT University, Ahmedabad, India

**Keywords:** Urban development, Water and sanitation, Water security, Performance assessment

## Abstract

Gujarat has made important strides to ensure that most parts of the state become water secure. In 2005, Gujarat was one of the few states that recognized that its urban areas were its ‘engines of growth’ and made significant investments in urban infrastructure. A state-wide water supply grid was constructed to transfer inter-basin water from perennial surface water sources to water-scarce areas. While these schemes have improved household-level access to municipal water supply, service levels have not improved. In this paper, we argue that along with infrastructure creation, there is a need to focus on monitoring, operation and maintenance of existing system and improving efficiency. We analyze information available from the performance assessment system (PAS) setup by the CEPT University for monitoring of urban services in India. It has annual information of water service delivery in all the urban areas of Gujarat from 2010. We assess urban drinking water supply on three key aspects: equity, service quality and financial sustainability. We also identify a few key intervention areas related to increased accountability, efficiency and equity in delivery of water supply services.

## Introduction

The ‘Gujarat model of development’ is known for its focus on economic development. It is also known that Gujarat was among the first states in the country to recognize that ‘cities are engines of development.’ The Government of Gujarat had declared the year 2005 as the ‘urban year’ to bring focus to urban development issues in the state and invest in urban infrastructure. The vision was widened and carried forward in the framework of the 'Swarnim Jayanti Mukhya Mantri Shaheri Vikas Yojana' (Chief Minister's Urban Development Scheme). It had an initial outlay of Rs. 7000 Crores to help realize the urban vision. These funds were used for creating infrastructure such as roads, streetlights, city transport services, water supply and sewerage systems. Subsequently, the outlay was increased to Rs. 18,000 Crores over the next 5 years. In addition, several state-level initiatives were also launched including the Nirmal Gujarat Shauchalaya Yojana for toilet coverage, liquid waste management for wastewater reuse and municipal solid waste management Project.

Gujarat’s focus on economic development has yielded results. Gujarat’s average annual per capita income at current prices in 2018–2019 is Rs 1,97,447, which is 56% higher than the national average and shows an increase of 14.1% over the previous year (Directorate of Economics and Statistics 2020). Along with the rise in income, Gujarat has done well to reduce urban poverty over the past decades. Poverty declined sharply in urban Gujarat from 20% in the year 2005 to 10% in the year 2012 (World Bank 2017). Rise in income and reduction in urban poverty have implications on demand for better urban services.

Despite high economic growth, a key challenge for cities in Gujarat has been provision of drinking water. 'In the year 2000, the Government of Gujarat (GoG) had planned a state-wide water grid to connect 75% of the state’s approximately 60 million urban and rural residents to drinking water sourced from the Sardar Sarovar Dam on the Narmada River. This has been the Government of Gujarat’s primary response to the issue of water scarcity for domestic and industrial use, replacing local dam—and groundwater—based schemes across the state.' (Luxion 2017). Mehta and Mehta (2011) had showed that with water from the Narmada River reaching the interiors of Kutch, North Gujarat and Suarashtra, and the Government of Gujarat’s priority to water sector, there is increased drinking water security in the state. However, as we argue in this paper, with significant investments in urban water infrastructure and increased access, the service quality—measured in quantum of water available and hours of supply has improved only marginally. More attention is also needed on efficiency of service delivery, measured as non-revenue water, thus suggesting that a greater focus is needed on improving local water management practices.

## Performance assessment system of water supply service delivery

Conventionally, assessment of water supply service delivery has been done using performance benchmarking frameworks. Assessment of water and sanitation services in developing countries, using such benchmarking frameworks, has been developed by various international agencies and a variety of lead institutions. This experience provides a rich basis for drawing lessons for assessment of water service delivery in developing countries. Some notable efforts have been made by the American Water Works Association (AWWA), the International Water Association (IWA) and the International Benchmarking Network for Water and Sanitation Utilities (IBNET) of the World Bank (see for example, Alegre et al. 2000, 2006; Matos et al. 2003; Cabrera et al. 2011; Berg and Danilenko 2011). See also a review of benchmarking approaches by Mehta et al. (2013). However, their use in the Indian context poses serious challenges as water supply services in Indian cities are generally intermittent, often unmetered and a large number of poor consumers who depend on shared connections (Vavaliya et al. 2016).

The initial Indian efforts related to benchmarking were mostly one-time efforts with the sole purpose of creating awareness about benchmarking to assess the status of urban water supply and sanitation (see for example, MoUD 2007; NIUA 2005). For benchmarking to result in service performance improvement, it pre-supposes that data used for performance measurement are available on a regular basis and are reliable. It also needs to recognize and incorporate the ground realities of cities in India.

It was in this context that the performance assessment system (PAS) for urban water supply and sanitation services was set up by the CEPT University. Access to water and sanitation services in urban India was widespread, but little was known about service levels and quality and about service coverage for poor households. The lack of reliable and updated information on these services often led to misallocation of resources. Such information gaps on service performance often resulted in an undue focus on building new infrastructure without adequate improvements in service quality. New investments often failed to improve the level and quality of service. For example, despite the Rs 50,000 Crores (US$7 billion) investment in water and sanitation during 2005–2010, under the Jawaharlal Nehru National Urban Renewal Mission (JNNURM) of the Government of India, little is known about how this investment improved service levels in cities.

In July 2008, CEPT University in Ahmedabad setup a performance assessment system (PAS) for urban water supply and sanitation (UWSS) in the two states of Maharashtra and Gujarat under a project funded by the Bill and Melinda Gates Foundation. The PAS project covered all 400 urban local governments in these two states. It worked with state and local governments to develop a reliable and sustainable system for assessing urban water and sanitation services. The indicators for performance measurement were developed through studies and stakeholder consultations at the state level and across cities. The performance monitoring included setting up appropriate systems at state level, with annual information, detailed analysis of indicators and documentation of good practices. A dedicated web platform (www.pas.org.in) was created to host this information. It enabled urban local bodies (ULBs) to track their performance and compare with peers. It is also aligned with the service-level benchmark (SLB) framework of the Government of India.

From 2010 onward, performance monitoring of urban services was also linked to the performance-linked grants provided by the 13th and 14th Finance Commissions for Urban Local Bodies (ULBs). The PAS portal is currently being used by over 900 cities in six Indian states—Gujarat, Maharashtra, Chhattisgarh, Telangana, Jharkhand and Assam—as a self-assessment tool. It helps annual tracking of their performance on water supply and sanitation services.

As per the Census 2011, majority of households (86%) in the state of Gujarat state were using tap water that is higher than the national average of 71% in urban areas (Census 2011, HH 6). The analysis presented in this paper is from the information available on PAS platform for piped water supply services provided by cities of Gujarat state. PAS online module is used as a self-assessment tool for municipal water supply services. Non-municipal water service is not covered. The analysis presented in subsequent sections is based on the information on the PAS platform. It must be noted that this information is provided by urban local bodies (ULBs) and related to municipal supply. It does not take into account ‘private’ sources of drinking water, e.g., wells and borewells. We have used two time periods—2010 and 2019—to show how water service delivery has changed in Gujarat over this decade. Although the PAS platform has a significant amount of information, we have used only a few key performance indicators to highlight how the situation has changed over this time period.

## Urbanisation in Gujarat

With an urban population of 25.7 million in 2011, which is 42.6% of the total state population, Gujarat ranks among the most urbanized states of India. The state registered a decadal urban population growth of 35.8% between 2001 and 2011. It is projected that Gujarat’s urban population will reach 45 million in 2036, at 55% of total population. This near doubling of urban population is likely to pose serious challenges in ensuring drinking water security to all (Table 1).

**Table 1:** Urbanization and growth trends *Sources*: Census of India (2011) and MoHFW (2018)

Year	Gujarat			India		
	Urban population (in Million)	Urban to total population (%)	Decadal growth rate (%)	Urban population (in Million)	Urban to total population (%)	Decadal growth rate (%)
1961	5.3	25.7	19.6	78.9	18.0	26.4
1971	7.5	28.1	41.1	109.1	19.9	38.3
1981	10.6	31.1	41.5	159.5	23.3	46.2
1991	14.2	34.5	34.3	217.6	25.7	36.4
2001	18.9	37.3	32.9	286.1	27.8	31.2
2011	25.7	42.6	35.8	377.1	31.2	31.8
2036	44.9	55.1	–	603.7	39.7	–

Gujarat’s urban population is also concentrated in a few cities. Nearly 50% of the urban population resides in four cities: Ahmedabad, Surat, Vadodara and Rajkot. These cities are envisaged as ‘Cities of Excellence.’^1^ Further, nearly two-thirds of Gujarat’s urban population resides in the eight Municipal Corporations (MCs) (Table 2).

**Table 2:** Distribution of urban population in different classes of ULBs in Gujarat *Source*: PAS 2019, from: www.pas.org.in

Category of ULBs	Number of ULBs	Population in 2019 in Million	% of total urban population
Municipal Corporation	8	19.1	65.0
Class A (greater than 100,000 population)	22	4.5	15.0
Class B (50,000–100,000 population)	34	2.8	9.0
Class C (25,000–50,000 population)	62	2.4	8.0
Class D (less than 25,000 population)	44	1.0	3.0
Total	170	29.8	100

## Assessing access to drinking water supply

The Joint Monitoring Programme (JMP) of the United Nations Children's Fund (UNICEF) and the World Health Organization (WHO) monitors the sustainable development goal 6, related to water supply and sanitation. It reviews drinking water services with respect to accessibility, availability and quality of the main source used by households for drinking, cooking, personal hygiene and other domestic uses. It defines safely managed drinking water service as the one that meets three criteria of: (i) It should be accessible on premises, (ii) water should be available when needed and (iii) the water supplied should be free from contamination (JMP 2019). Our analysis of drinking water availability in urban Gujarat is also assessed through access to piped water on premise, quality of water and duration of supply. This is captured in various related service delivery indicators described on the PAS portal.

### Access

In the PAS framework, access is measured as households connected to the water supply network with a private (not shared) service connection. In 2010, 79 of urban households in Gujarat had tap water within premise. There is a significant increase in access to piped water supply, as shown in Table 3. In 2019, 89% of urban households in Gujarat had tap water connections at home.

**Table 3:** Classwise coverage of water supply connections: 2010 and 2019 *Source*: analysis based on data by the urban local governments in Gujarat as reported on the performance assessment system (PAS) Portal

Coverage of water connections by class of city (%)		
Class of city (population range)	Year 2010	Year 2019
Municipal Corporation	87	96
Class A (>100,0000	63	72
Class B (50,000–100,000)	72	78
Class C (25,000–50,000)	68	77
Class D (<25,000)	68	83
State	79	89

However, the access is not uniform across cities of different sizes. Access in large cities in Municipal Corporations is generally higher than in the smaller towns. However, it was surprising to see that class D towns had higher access levels. This may be due to the fact that these towns were earlier classified as rural areas and may have benefitted from the piped water supply schemes in rural areas. Some cities, such as Municipal Corporation of Bhavnagar, Class A cities of Nadiad and Patan and Class B cities of Dabhoi and Kadi, have reached 100% coverage or universal access to piped water supply connections. These cities have good local governments that have managed to ensure universal coverage. On the other hand, many cities, such as Junagadh Municipal Corporation, and class A cities of Anand, Botad, Gandhidham, Mehsana, Morbi and Palanpur have less than 70% of their households with piped water supply. This is possibly due to the fact that many of these cities have extended their boundaries in the last decade and a large number of households in the extended parts of the city are yet to receive water supply connections.

In 2017, urban development department divided into the state in six divisions, with each division headed by a Regional Commissioner, Municipalities (see Appendix for a map and list of districts in each division). We have assessed water services at division level and, where necessary, provided examples of cities. Full analysis is available at www.pas.org.in. Table 4 shows that cities in Ahmedabad and Surat divisions have higher coverage as compared to other divisions. On the other hand, divisions in Suarashtra and Kutch—Bhavnagar and Rajkot divisions—have lower access to piped water supply. These are water scarce divisions, and Narmada water has reached these divisions only in 2015.

**Table 4:** Divisionwise coverage of water supply connections: 2010 and 2019 *Source*: analysis based on data by the urban local governments in Gujarat as reported on the performance assessment system (PAS) Portal by urban local governments

Coverage of water connections by divisions (%)		
Division	Year 2010	Year 2019
Ahmedabad	89	94
Bhavnagar	72	77
Gandhinagar	78	83
Rajkot	65	80
Surat	82	93
Vadodara	69	89

### Access to water supply in slum areas

As per 2019 data from urban local governments reported on the PAS portal, around 11% urban population in Gujarat lives in slums. There are 3400 slum settlements in 157 cities. For the state as a whole, water supply network covers 95% of slum areas. In these ‘covered slums,' 79% of households have access to own tap water supply, as compared to 86% for all residents of the cities.^2^ Presence of water supply infrastructure is not a challenge in slums, but providing last-mile household connectivity is a major bottleneck. 'Simplifying connection procedures and reducing connection costs can go a long way in facilitating urban poor to access formal service systems' (EIB and WaterAid India 2018). Some cities such as Ahmedabad in Gujarat have demonstrated special initiatives to enable households in slums to overcome legal issues to access water supply services. Given this experience, universal access to municipal water supply services in slums in Gujarat is a possibility over the coming years.

## Water supply service levels, efficiency and cost recovery

In water supply performance assessment, the service level is measured by the per capita supply of water and by continuity of this water supply. Efficiency is measured as extent of non-revenue water and extent of metering. Financial sustainability is measured through operation and maintenance cost recovery.

### Quantity of municipal water supply

Urban areas in Gujarat have seen a near doubling of quantity of municipal water supply during the last decade. In 2010, 3422 million liters per day (MLD) of water was supplied in urban areas. This had increased to 5222 MLD by 2019. This increase was mainly through bulk water purchase by cities, which increased from 2382 MLD in 2010 to 3978 MLD in 2019. It was mainly due to availability of Narmada water to cities in North Gujarat, Saurashtra and Kutch division. Municipal water supply through ground water source has increased only marginally from 808 MLD in 2010 to 988 MLD in 2019 despite a significant rise in urban population. There are still 43 ULBs that continue to be entirely dependent on ground water, though this number has come down from 63 ULBs in 2010 that were dependent solely on the ground water sources. Thus, a few ULBs have decreased their dependency on ground water as a source for municipal water supply in the last decade. This is also corroborated by NSS studies. As per the National Sample Survey (NSS) round 69 (July to December 2012) and round 76 (July to December 2018), tube well/borewell share in drinking water has declined from 11 to 5.3% among urban households in Gujarat.

With an increase in total quantum of water supplied, per capita water supply (lpcd) at the consumer end has also increased in the state over the last decade. This increase is observed across all divisions (Table 5). However, there are differences across size class of cities. The Municipal Corporations have a much higher per capita supply as compared to the smaller towns. The smaller towns still do not have the recommended benchmark level of 135 lpcd (MoUD 2008).

**Table 5:** Classwise per capita water supply: 2010 and 2019 *Source*: analysis based on data by the urban local governments in Gujarat as reported on the performance assessment system (PAS) Portal by urban local governments

Liters per capita supply (lpcd)		
Class	Year 2010	Year 2019
Municipal corporation	117	146
Class A	79	105
Class B	83	101
Class C	73	93
Class D	73	111
State	102	130

### Duration of water supply

From consumer perspective, water security implies availability of acceptable quantity and quality of water when needed. Despite the increase in quantity of drinking water supply, all cities in Gujarat have only intermittent water supply, with water being supplied only for a few hours every day. In Gujarat, the average duration of water supply to consumers is two hours per day and the average number of water supply days in a month is 23 days. There has been no major change in supply hours over the past nine years. While 103 ULBs supply water every day, 44 ULBs supply water every alternate day and 19 ULBs supply water every third day or less. Cities have still not achieved the water security from consumer perspective where they still have to pump and store water, often for days.

Intermittent water supplies are also susceptible to contamination due to the negative pressure conditions after supply hours, which allow the ingress of contaminated groundwater or wastewater through leaky pipes and joints (WHO 2014). It can also lead to pipe corrosion and failure caused by exposing the system alternately to water and to air. More water is typically used in intermittent water supply because even though it is intended as a response to water shortage, consumers need to store water between supplies. In intermittent supply, consumers adopt expensive coping strategies that include installing underground storage tanks, suction pumps on water mains or overhead tanks, boiling water or using household filters (WSP 2010). Those, who cannot afford pumping and storage, have to use and tend to throw away stored water, which causes water wastage. Often, the taps are left open during the entire supply time resulting in wastage of water. These issues require attention. It is common to find that middleclass households and the rich are able to address this by having overhead tank facilities in their buildings. However, the poor face difficulties due to the lack of space for water storage.

Thus, despite an overall increase in supply of water and increased per capita supply, there are problems with distribution across zones and across income class in cities. While per capita supply has increased, the duration of water supply has not increased. In very few cities, days and hours of water supplied have improved over the last decade (Table 6). Out of 64 ULBs which do not supply water daily, 52 cities supply more than 70 lpcd to consumers. This suggests that while these cities have adequate quantity of water, they have a problem with their distribution network. With proper pressure management system, these cities may be able to supply water every day.

**Table 6:** Divisionwise days of water supply at consumer end *Source*: analysis based on data by urban local governments in Gujarat as reported on the performance assessment system (PAS) Portal by urban local governments

Divisions	Year 2019				Year 2010			
	Daily	Alternate days	<15 days	Total cites	Daily	Alternate days	<15 days	Total cites
Ahmedabad	19	7	2	28	18	4	5	27
Bhavnagar	8	16	5	29	6	17	6	29
Gandhinagar	26	5	0	31	24	5	1	30
Rajkot	8	12	12	32	5	18	9	32
Surat	23	0	0	23	21	0	0	21
Vadodara	22	5	0	27	22	5	0	27
Total cities	106	45	19	170	96	49	21	166

Cell values are number of cities

### Non-revenue water

In the discussion of water supply management, non-revenue water generally emerges as one of the most important parameters to assess efficiency. Non-revenue water (NRW), as the name suggests, is the water for which the water supply agency does not get any revenue. This can occur through physical losses from leakages through broken pipes and improper joints. This is caused by poor operations and maintenance, and the lack of active leakage control. However, it can also result from theft of water through illegal connections or by extra withdrawal of water by installing illegal pumps.

One of the major challenges for ULBs is the high level of non-revenue water. If a large proportion of water supply is lost, the per capita water supply at the consumer end reduces significantly. In addition, this lost water yields no revenue and losses make it harder to keep water tariffs at a reasonable and affordable level. Reducing NRW can significantly improve the service level. It may also enable cities to supply water daily and for a greater number of hours.

One of the major difficulties in measuring non-revenue water (NRW) is the complete lack of meters to measure water supply in the state. The absence of meters is observed not only at consumer end but also at the source of supply and distribution stations in the city. Only four Municipal Corporations have water meters at the consumer end for a few connections. Only 43 ULBs have reported flow meters installed at water treatment plants, and 34 ULBs have flow meters installed at water distribution stations. With the absence of such meters, it is almost impossible to measure NRW properly. Therefore, none of the reported NRW value in Table 7 is reliable. They are indicative of the efforts that ULBs have made to reduce the NRW. Proper NRW assessment is important for moving away from the intermittent supply to a 24*7 water supply in Gujarat cities. This was envisaged as a key reform in many Government of India reform programs, though it has failed to take off.

**Table 7:** Division non-revenue water (NRW) in water supply system: 2010 and 2019 *Source*: analysis based on data by the urban local governments in Gujarat as reported on the Performance assessment system (PAS) Portal by urban local governments

Non-revenue water (%)		
Division	2019	2010
Ahmedabad	21	29
Bhavnagar	19	28
Gandhinagar	20	24
Rajkot	20	35
Surat	20	35
Vadodara	24	27

A preliminary water audit study by Sapient Techno Consultants for a city in Gujarat found the NRW level of 46% (Sapient 2010). Following this, various studies conducted in Gujarat suggest NRW levels are much higher than those reported by ULBs. This suggests that while adequate quantity of Narmada water is made available to cities, all of it does not reach the consumers. It is important that just as financial audit is carried out each year for cities, there should also be a water audit done each year.

However, very few cities in Gujarat have been conscious about reducing water losses. The Municipal Corporations in Gujarat have now installed Supervisory Control and Data Acquisition (SCADA) systems in transmission network (from sources to distributions stations). These are computerized systems for gathering and analyzing real-time data on volume of water passing through the system at various points. This enables municipal engineers to check water flows in the system and identify leakages. By installing SCADA, Rajkot Municipal Corporation has reduced water losses through leakages in transmission network. This suggests that other cities can also achieve efficiency through regular monitoring of flows. However, to assess and reduce NRW, it will be essential to install consumer water meters, and introduce regular monitoring.

### Cost recovery

National policy expects that ULBs should recover all the operating costs of water supply. The service-level benchmark (MoUD 2008) defines cost recovery as 'the total operating revenues expressed as a percentage of the total operating expenses incurred in the corresponding time period. Only income and expenditure of the revenue account must be considered, and income and expenditure from the capital account should be excluded.'

There have been a number of studies on cost recovery of urban water and sanitation. A WSP (2011) study concluded that 'water services in more than half of the 23 cities analyzed here are suffering substantial operational losses. None of these ULBS met their revenue potential, and most of them fail to cover their operational costs by up to 80%. It is true that low tariffs are a reason for this: Tariffs are mostly based on estimates rather than quantified costs and margins.' Singh et al. (2005) state that 'given the high level of per capita investment and the history of government-subsidized services, full-cost pricing of water services has yet to take hold in India. As a result, it remains broadly under-priced leading to public perception that water is 'free''.

On an average, cities in Gujarat spend Rs 1679 per household on operation and maintenance (O&M) costs of drinking water supply in the cities. However, they collect only Rs. 1188 per household through fixed water charges. The Municipal Corporations recover 85% of their O&M costs, whereas municipalities recover only 35% of their O&M costs. In Gujarat, 121 ULBs (70% of ULBs) report cost recovery of less than 50%.

Mathur and Thakur (2003) state that 'most water supply entities—be these the Public Health Engineering Departments (PHED), state- or city-level water boards, or municipal governments, run at a loss, and cover the loss—defined as the gap between revenues from the sale of water and cost of water provision—from government subsidies and accelerated depreciation of capital. The result is a low-level equilibrium: low tariff, poor services and constraints on access, especially of poor households. While the need for appropriate pricing of urban water has been long stressed and is widely recognized as central to broader urban sector reforms, what constitute water price reform remains an elusive and emotive issue.”

Nearly 40% of operation and maintenance cost of water supply is for electricity charges. ULBs also pay for purchase of bulk water from Narmada and other irrigation projects. The Gujarat state government decides bulk water charges for domestic, industrial and agriculture uses. For example, Narmada water is available to municipalities at Rs. 4 per Kilo Liter (KL) and to Municipal Corporations at Rs 6 per KL. These charges are fixed for the entire state irrespective of topography or distance. These charges should be viewed in light of the fact that the real cost of Narmada water is Rs 85 per KL (Hirway and Goswami 2008).

Tariff is the set of prices, charges, and taxes used to generate revenue. A well-designed tariff enables financially sustainable service delivery and encourages users to avoid wasteful consumption. However, in the absence of metered connections at household level, most ULBs in the state have fixed water supply charges ranging from Rs. 150 to Rs. 1400 per connection per year. Such fixed water charges are regressive as consumers pay the same amount irrespective of the quantum of water consumed. There is, thus, an inherent subsidy to the non-poor households, as they have the ability to pump and store water during supply hours. A few Municipal Corporations have attempted to address this problem by linking the water charge to property tax assessment. For example, in Ahmedabad, the water charges are 30% of the property tax. This makes it less regressive, but still not a fair pricing of water as it is delinked from consumption. In Gujarat, without metered supply, it is not possible to use water tariffs as a means for water demand management and promote efficient and equitable use of water.

The Government of Gujarat had issued guidelines in 2010^3^ that water supply charges should be at least Rs 600 per year per property. This guideline has had an adverse impact in a few cities as cities that were charging more for water prior to the issuance of this guideline, had to reduce their water charges due to political pressure. Also, these guidelines did not provide any indexation with rising costs; hence, the water charges have remained unchanged for many ULBs. As a result, 135 ULBs (80% of all ULBs) have continued to levy a fixed water charge of Rs. 600, without any change over the last ten years.

## Conclusion

Gujarat has made significant strides in economic development, increase in employment and alleviation of poverty. It is also one of the first states in India to focus on urban areas. This would suggest that infrastructure and services in cities in Gujarat would be commensurate with economic growth. There is a significant investment in water infrastructure. It is estimated that Rs 47,000 Crores was spent on Narmada project (DNA 2015). Water from the Narmada canal has reached remote parts of the state. This has alleviated water crisis that cities in North Gujarat and Saurashtra faced during summer months. 'In 2002, emergency arrangements to meet water shortages were replaced with a longer-term strategy: the construction and management of the state-wide water supply grid. This scheme moves toward connecting 47 million people to safe, potable water supplies. It has also positioned Gujarat as a pioneer in India in terms of moving toward water security and conservation, a policy choice that has boosted economic growth and made important strides toward human development.' (Biswas-Tortajada 2014).

Having achieved water security in urban areas, it is now important for Government of Gujarat and the urban local governments to focus on improving water management. This would require ensuring proper distribution of available water to all parts of the state. Smaller cities that receive less than 70 lpcd of water need to be provided more water. In addition, as we have seen, higher quantity of water does not necessarily translate into higher level of service to consumers. The high level of non-revenue water (NRW) needs to be reduced. The lack of meters at various points in water supply system makes it difficult to realistically assess NRW and move toward efficient water management. The NRW assessment is also important for moving away from the intermittent supply to a 24*7 water supply in Gujarat cities. Just as a financial audit is carried out each year for cities, there should be a water audit done each year. This was envisaged as a key reform in many Government of India reform programs, though it has failed to take off in Gujarat.

Gujarat needs to adopt a proper water tariff policy. Volumetric water tariff is accepted as a norm globally. Rising block tariff, as used for domestic electricity providers, needs to be adopted for drinking water supply. Consumer-level meters will be needed to implement volumetric tariff. Only then, ULBs in Gujarat can achieve the benchmark of 100% cost recovery of operating expenditure.

Cities in Gujarat have increased coverage of water supply services even in slum areas. However, our analysis highlights that the access to municipal water service is not uniform across all cities. Access in Municipal Corporations is generally higher than in the municipalities. While the total quantum of water supplied in cities has increased, the duration of water supply and the number of days of water supply have not changed significantly over the ten-year period. If we look at the water security from consumer perspective, then they still have to pump and store water, often for days. In May 1986, potable water was transported over 200 kms by train to Rajkot to save the city from evacuation due to the paucity of drinking water. This led to planning of a state-wide water grid to ensure drinking water to all rural and urban settlements in Gujarat. Gujarat has come a long way in improving water security to its residents. The next task is to ensure that the city residents receive a higher level of drinking water service. The safely managed drinking water service as defined by WHO-UNICEF is the one that meets three criteria of: (i) It should be accessible on premises, (ii) water should be available when needed and (iii) the water supplied should be free from contamination. Cities in Gujarat should plan to meet these criteria.

## Appendix 1: List of divisions and districts

**Table:** 

Division	Districts
Ahmedabad	Ahmedabad, Kheda, Surendranagar, Botad
Gandhinagar	Patan, Banaskantha, Mehsana, Sabarkantha, Aravalli, Gandhinagar
Vadodara	Panchmahal, Mahisagar, Dahod, Vadodara, Anand, Chotta Udaipur
Rajkot	Katch, Jamnagar, Devbhoomi Dwarka, Morbi, Rajkot, Porbandar
Bhavnagar	Amreli, Gir Somnath, Junagadh, Bhavnagar
Surat	Tapi, Narmada, Navsari, Bharuch, Valsad, Surat

## Appendix 2: Map showing divisions and districts


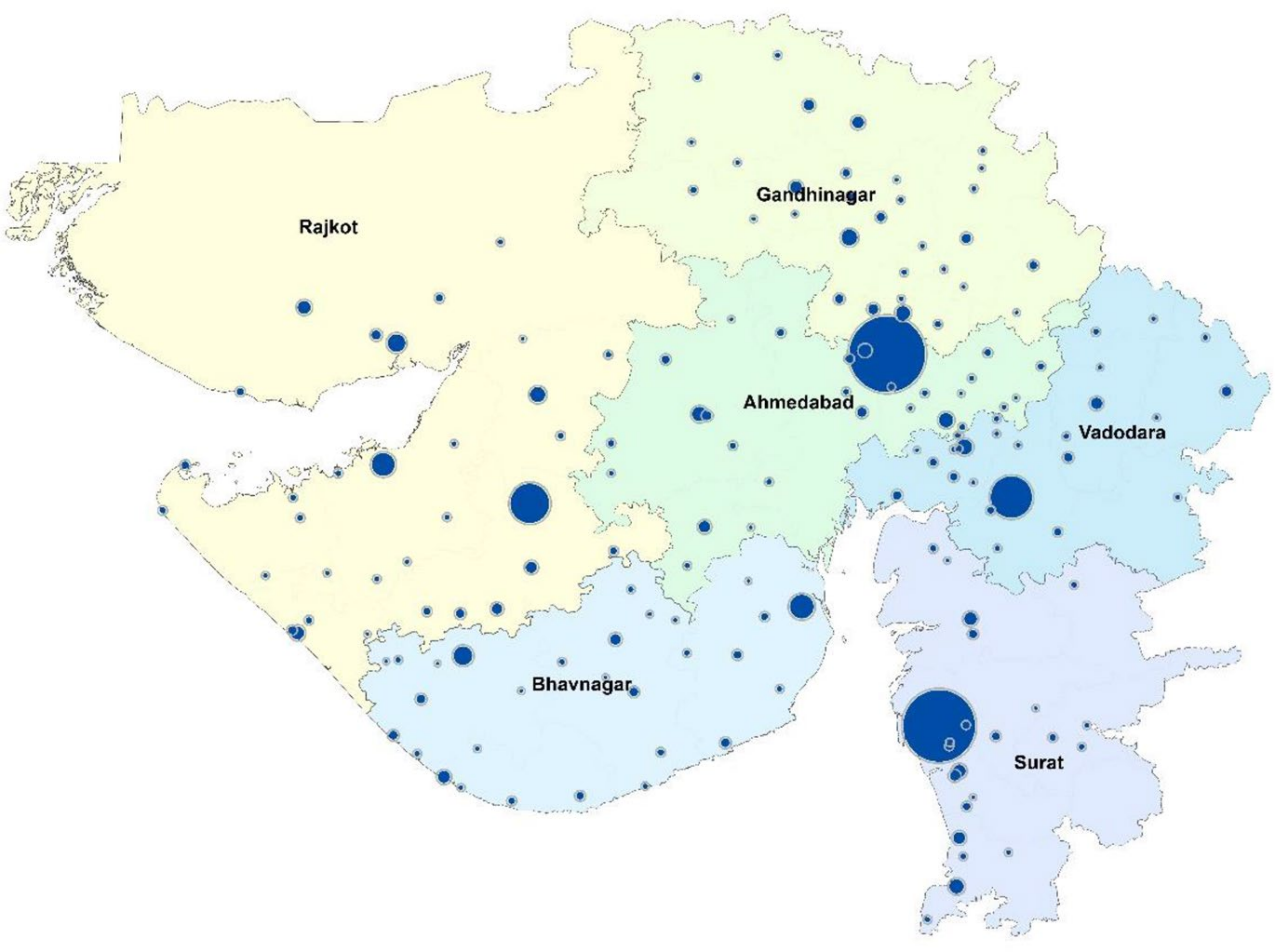


## Footnotes

1 Gujarat’s vision for urban sector, https://www.gidb.org/urban-infrastructure-gujarats-vision.

2 Average value of water supply connections coverage in city and slum is calculated based on 153 cities data (information of water supply connections coverage in slum is not available for four cities, Gandhinagar, Surat, Dakor and Maliyamiyana, and 13 cities reported no slum within city boundary; Bopal Ghuma, Dhrangadhra, Kadi, Sanand, Babra, Bagasra, Jafrabad, Kansad, Lunavada, Sutrapada, Chanasma, Vadali, Vanthali).

3 Government of Gujarat’s guidelines on minimum water tariff (GR No 4510/CF-1/M on 21/4/2010).

## References

[CR1] Alegre H, Baptista JM, Cabrera E, Cubillo F, Duarte P, Hirner W, Merkel W, Parena R (2006) Performance indicators for water supply services. In: Manual of best practices, 2nd ed. IWA Publishing House, London

[CR2] Alegre H, Hirner W, Baptista J, Parena R (2000) Performance indicators for water supply services. In: Manual of best practices, 1st ed. IWA Publishing House, London

[CR3] Biswas-Tortajada A (2014). The Gujarat state-wide water supply grid: a step towards water security. Int J Water Resour Dev.

[CR4] Berg CV, Danilenko A (2011). The IBNET water supply and sanitation performance blue book: the international benchmarking network.

[CR5] Cabrera E, Dane P, Haskins S, Theuretzbacher-Fritz H (2011) Benchmarking water services: guiding water utilities to excellence. In: Manual of best practice. IWA Publishing, London

[CR6] Census of India (2011) Main source of drinking water 2001–2011. Data Product No. 00-015-2011-Cen-Data Sheet (E). https://www.censusindia.gov.in/2011census/hlo/Data_sheet/India/Drinking_Water.pdf

[CR7] DNA (2015) https://www.dnaindia.com/india/report-rs-47202-crore-spent-on-narmada-project-gujarat-government-2068073

[CR8] Directorate of Economics and Statistics (2020) Socio economic review: 2019–2020. Gandhinagar. https://gujecostat.gujarat.gov.in/sites/default/files/Bud-Guj_2020final.pdf

[CR9] EIB-Exhibitions India Group and WaterAid (2018) State of urban water supply in India. https://www.wateraidindia.in/sites/g/files/jkxoof336/files/state-of-urban-water-supply.pdf

[CR10] Hirway I, Goswami S (2008). Functioning of the drinking water component of the Narmada pipeline project in Gujarat. Econ Political Weekly.

[CR11] JMP-Joint Monitoring Programme (2019) Progress on household drinking water, sanitation and hygiene 2000–2017. Special focus on inequalities. United Nations Children’s Fund (UNICEF) and World Health Organization, New York

[CR12] Luxion M (2017). Nation-building, industrialisation, and spectacle: political functions of Gujarat’s Narmada pipeline project. Water Altern.

[CR13] Mathur OP, Thakur S (2003). Urban water pricing: setting the stage for reforms.

[CR14] Matos R, Cardoso A, Ashley R, Duarte P, Molinari A, Schulz A (2003) Performance indicators for wastewater services. In: Manual of best practice. IWA Publishing, London

[CR15] Mehta M, Mehta D, Parthasarathy R, Dholakia RH (2011). Urban drinking water security and sustainability in Gujarat. SardarSarovar project on the river Narmada: impacts so far and ways forward, Chapter 25.

[CR16] Mehta M, Mehta D, Immanuel A (2013) A review of performance benchmarking: urban water supply and sanitation, performance assessment system (PAS). CEPT University, Ahmedabad. https://pas.org.in/Portal/document/ResourcesFiles/pdfs/Review of Performance benchmarking Report.pdf

[CR17] MoHFW-Ministry of Health and Family Welfare, Government of India (2018) Report of the Technical Group on population projections. Population projections for India and States 2011–2036, November 2018.

[CR18] MoUD (2008) Handbook of service level benchmarking. Ministry of Urban Development, Government of India

[CR19] MoUD Ministry of Urban Development and ADB-Asian Development Bank (2007) Benchmarking and Data Book on Water Utilities in India

[CR20] NIUA-National Institute of Urban Affairs (2005) Status of water supply, sanitation and solid waste management in Urban Areas. Report for the CPHEEO and MoUD, Government of India

[CR21] NSS 69th Round (2014) Ministry of statistics and programme implementation, Government of India. Drinking water, sanitation, hygiene and housing condition in India. NSS Report No. 556 (69/1.2/1)

[CR22] NSS 76th Round (2019) Ministry of statistics and programme implementation, Government of India. Drinking water, sanitation, hygiene and housing condition in India. NSS Report No. 584 (76/1.2/1)

[CR23] Sapient (2010) Preliminary water audit: estimation of water losses and strategy for loss reduction city of Kalol, Gujarat, India. https://www.pas.org.in/Portal/document/PIP Application/Kalol Water Audit Report.pdf

[CR24] Singh MR, Upadhyay V, Mittal AK (2005). Urban water tariff structure and cost recovery opportunities in India. Water SciTechnol.

[CR25] Vavaliya J, Bhavsar D, Kavadi U, Mohammad M (2016) Online performance assessment system for urban water supply and sanitation services in India. Aquat Proced 6: 51–63. https://www.sciencedirect.com/science/article/pii/S2214241X16300074

[CR26] WHO-World Health Organization (2014) Water safety in distribution systems. WHO Document Production Services, Geneva. https://www.who.int/water_sanitation_health/publications/Water_safety_distribution_systems_2014v1.pdf

[CR27] World Bank (2017) Gujarat: poverty, growth, and inequality (English). India state briefs. World Bank Group, Washington, DC. https://documents.worldbank.org/curated/en/933681504004310148/Gujarat-Poverty-growth-and-inequality

[CR28] WSP-Water and Sanitation Programme (2010) Field note on The Karnataka urban water sector improvement. World Bank. https://www.wsp.org/sites/wsp/files/publications/WSP_Karnataka-water-supply.pdf

[CR29] WSP-Water and Sanitation Programme (2011) Cost recovery in urban water services: select experiences in India Cities, World Bank. https://www.wsp.org/sites/wsp/files/publications/WSP-Cost-Recovery-Urban-Water-Services.pdf

